# Statin-Induced Triad of Autoimmune Myocarditis, Myositis, and Transaminitis

**DOI:** 10.1155/2021/6660362

**Published:** 2021-04-08

**Authors:** Muhammad Ajmal, Amitoj Singh, Saad Kubba, Michelle Hershman, Tushar Acharya

**Affiliations:** ^1^Division of Cardiology, Department of Internal Medicine, USA; ^2^Department of Medical Imaging, University of Arizona College of Medicine, Tucson, USA; ^3^Banner University Medical Center, Tucson, USA

## Abstract

Despite well-established cardiovascular benefits, statins have been associated with myopathic side effects ranging from myalgias to rhabdomyolysis and autoimmune necrotizing myositis. Statins have not been previously shown to cause myocarditis. Our case highlights this rare entity.

## 1. History of Presentation

A 70-year-old male presented to the emergency department (ED) with shortness of breath and generalized body aches associated with muscle weakness in all extremities. These symptoms dated back a few months; however, there was an acute decompensation in the past 1 week which led to the hospital visit. Shortness of breath was associated with orthopnea and chest discomfort. At the time of presentation to the ED, there was severe limitation of daily activities including inability to get out of bed, stand from a sitting position, and perform personal grooming. Presenting vitals were stable (pulse 84 beats per minute, blood pressure 134/60 mmHg). Physical examination demonstrated diffuse tenderness and objective weakness in all four extremities (3/5 strength). Reflexes were intact, and there were no focal neurological deficits. Cardiorespiratory examination demonstrated bibasilar crackles, an elevated JVP, and bilateral lower extremities pitting edema. An electrocardiogram demonstrated a normal dual-chamber function with ventricular pacing and atrial sensing.

## 2. Past Medical History

The patient had known diffuse nonobstructive coronary artery disease (CAD) (by coronary angiography). Other comorbidities included chronic kidney disease stage III and complete heart block status postdual-chamber pacemaker (implanted 1 year ago). His medications included atorvastatin 80 mg daily (6 months), metoprolol tartrate 25 mg twice daily, aspirin 81 mg, and glipizide 5 mg daily.

## 3. Differential Diagnosis

Clinically, the patient had signs and symptoms of a combined skeletal (proximal muscle weakness) and myocardial insult (acute heart failure with evidence of elevated filling pressures). While statins were suspected to at least be partially responsible for the clinical presentation, a unifying pathophysiology was sought and would require further testing. Subsequently, focused laboratory testing, imaging, and eventual pathological testing were performed as described below.

## 4. Investigations

Laboratory examination revealed rhabdomyolysis with acute kidney injury, transaminitis, and myocardial injury (elevated hs-troponin and NT-pro BNP). Rheumatologic workup yielded an abnormally elevated anti-HMGCR-Ab, which was suggestive of autoimmune necrotizing myositis with moderate significance (Table 1). Skeletal muscle biopsy (gastrocnemius) confirmed necrotizing myopathy and neurogenic atrophy.

**Figure 1:**
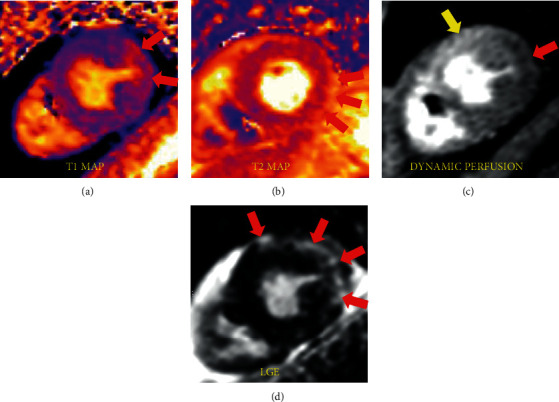
(a) Bright signal intensity on T1 map showing increased extracellular compartment due to inflammation/fibrosis. (b) Bright signal intensity on T2 map corresponding to inflammation/edema. (c) Normal (yellow arrow) and abnormal (red arrow) perfusion due to vascular injury/myocardial edema in the areas corresponding to (d) late gadolinium enhancement (LGE).

**Figure 2:**
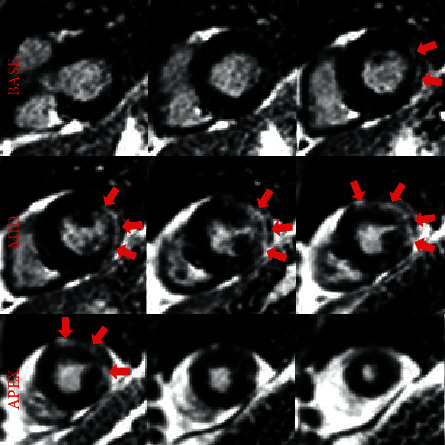
Late gadolinium enhancement (red arrows) in a midwall and epicardial distribution involving the inferolateral, anterolateral, and anterior walls consistent with myocarditis (short axis stack).

**Figure 3:**
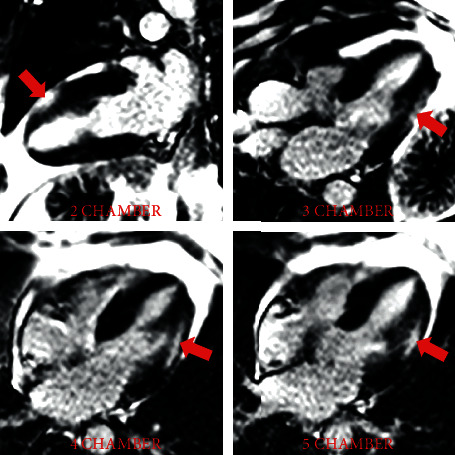
Late gadolinium enhancement (red arrows) in a midwall and epicardial distribution involving the inferolateral, anterolateral, and anterior walls consistent with myocarditis (long axes).

**Figure 4:**
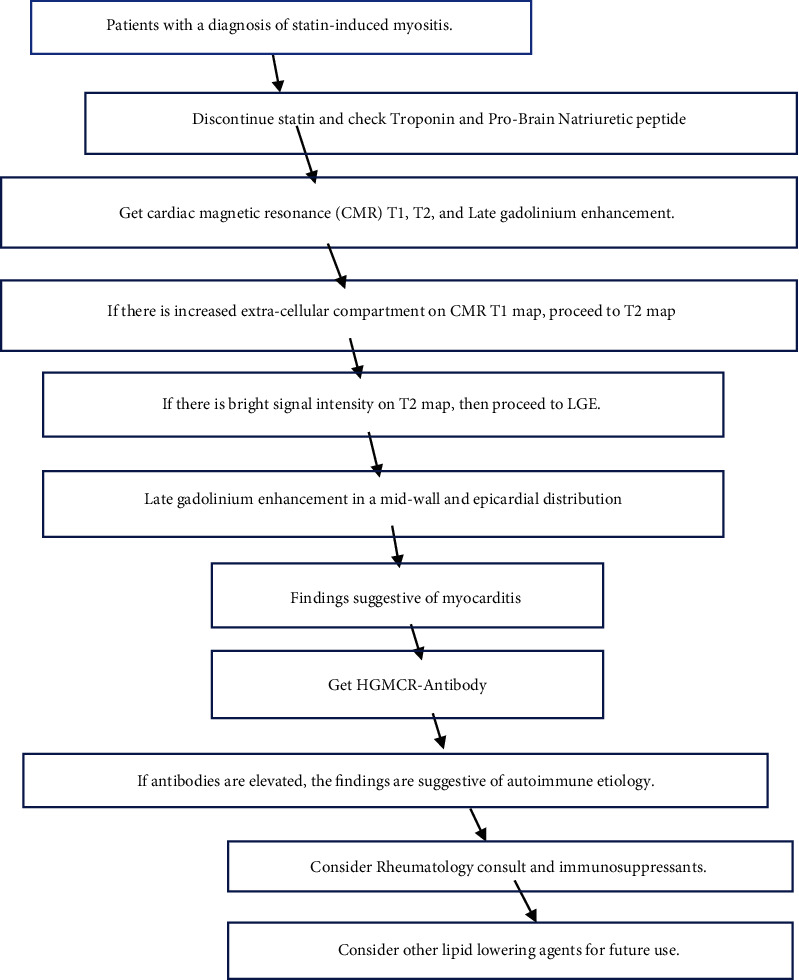
Algorithm for evaluating statin-induced myocarditis (LGE (late gadolinium enhancement)).

Magnetic resonance imaging (MRI) of the upper and lower extremities demonstrated diffuse muscular edema in keeping with acute myositis (Figure 1). Due to myocardial injury (elevated serum hs-cTnT and NT-pro BNP), cardiac involvement was suspected. An echocardiogram and subsequently a cardiac MRI were obtained. Echocardiography demonstrated normal biventricular size and function without regional wall motion abnormalities and no significant valvular abnormalities. Cardiac MRI confirmed normal biventricular size and overall LV function as seen on the echocardiogram. On tissue characterization, myocardial T1 was significantly elevated (Figure 1(a)) in the midanterolateral and inferolateral left ventricular walls suggesting expanded extracellular compartment due to edema/inflammation or fibrosis. T2 mapping images also demonstrated mildly increased values corresponding to these same areas in keeping with myocardial edema (Figure 1). On dynamic perfusion imaging, there was a resting perfusion defect in these areas, presumably due to perivascular edema or direct vascular injury. Late gadolinium enhancement imaging was diagnostic for myocarditis and showed enhancement in a midwall distribution involving the midantero- and inferolateral walls and in an epicardial distribution in the apical anterior wall (Figures 1(d), 2, 3). Our proposed algorithm for evaluating statin-induced myocarditis is shown in Figure 4.

## 5. Management

The initial suspicion for statin-induced myositis and rhabdomyolysis was confirmed with an elevated HMGCR antibody, abnormal MRI of extremities, and inflammatory myocyte necrosis on skeletal muscle biopsy. Atorvastatin was discontinued, and patient was resuscitated with intravenous normal saline. Close cardiac monitoring and electrolyte replacement were undertaken given acute myocarditis. Through the initial week of his hospitalization, the patient's renal function improved but neuromuscular weakness and pain continued. Once fulminant necrosis was confirmed on biopsy, given persistent skeletal weakness, immunosuppressive therapies were instituted. These included intravenous Solu-Medrol (125 mg daily × 3 days) and intravenous immunoglobulin (IVIG) (2 mg/kg IVIG × 1 dose). Despite this regimen, the patient remained clinically and chemically symptomatic (total CK and liver enzymes remained elevated). This prompted a trial of intravenous rituximab (1 g). Due to ongoing need for IV immunosuppression and slow improvement in muscle strength, the patient had a protracted hospital course lasting 4 weeks. Once clinically stable, he was discharged to a short-term rehabilitation facility. His laboratory work at discharge showed an improvement in CK (2815 U/L), AST (182 U/L), ALT (411 U/L), and ALP (172 U/L). He did not develop arrhythmias during the hospital stay.

## 6. Discussion

Despite its multiple, well-documented beneficial effects in atherosclerotic cardiovascular disease, statin use has been associated with side effects like muscle and liver injury [1, 2]. Myalgias have been commonly reported with statins but only 1 out of 10,000 patients develops objective muscle injury with CK elevation [3]. Most of these resolve spontaneously with statin cessation. In contrast, statin-induced autoimmune necrotizing myositis (ANM) is very rare but potentially life-threatening. It is diagnosed with antibodies to HMGCR and muscle necrosis on biopsy. ANM requires aggressive immunosuppression in addition to statin cessation [4–6].

Statin-induced myocarditis with or without associated autoimmune necrotizing myositis has not been reported in literature before. In fact, some reports suggest that statins may be helpful in treating myocarditis and postmyocarditis dilated cardiomyopathy [7]. This beneficial effect in myocarditis and particularly autoimmune myocarditis is thought to be due to anti-inflammatory/pleotropic effects of statins and possibly mediated by inhibition of antigen-presenting cells and lymphocytes leading to quiescence of an inflammatory surge and reduction in inflammatory biomarkers [8].

Contrary to these reports, our case represents a rare instance where statins can act as an offender and result in autoimmune myocarditis. Myocarditis is likely mediated by direct tissue injury not too different in its pathogenesis from the necrotizing myositis of the skeletal muscle. It is hypothesized that statin-induced overexpression of HMGCAR in genetically susceptible individuals may lead to autoimmunity against HMGCAR causing muscle injury. Since injured muscles produce more HMGCAR, this may perpetuate a cycle that may not be abated by discontinuation of statin and require immunosuppression [5].

Readers should familiarize themselves with this extremely rare adverse effect, especially in setting of elevated cardiac enzymes, which can be misinterpreted as acute coronary syndrome instead of myocarditis.

## 7. Follow-Up

The patient was an outpatient in the cardiology and rheumatology clinic 4-week posthospital discharge and continued to show clinical improvement (he could now walk without support). His steroid regimen is gradually being tapered over 6 months with close rheumatologic follow-up.

## 8. Conclusion

This is the first reported case of statin-induced myocarditis. Myocarditis in this patient is likely a part of the larger spectrum of statin-induced autoimmune necrotizing myositis, a very rare condition managed with aggressive immunosuppressants.

## 9. Learning Objectives

1. Recognize autoimmune necrotizing myositis and myocarditis as a rare complication of statin use
2. Diagnosis and management of this life-threatening myopathy

## Figures and Tables

**Table 1:** Laboratory findings.

Laboratory	Patient's results	Normal value
White blood cells	7.3 K/mm^3^	4-11 K/mm^3^
Hemoglobin	10.7 g/dL	13.5-17 g/dL
Platelets	230 K/mm^3^	130-450 K/mm^3^
Sodium	141 mmol/L	134-147 mmol/L
Potassium	5.3 mmol/L	5.3 mmol/L
Chloride	110 mmol/L	95-108 mmol/L
Bicarbonate	17 mmol/L	>19 mmol/L
BUN	67 mg/dL	8-25 mg/dL
Creatinine	1.8 mg/dL	<1.5 mg/dL
Anion gap	14	
AST	716 U/L	<50 U/L
ALT	907 U/L	<60 U/L
ALP	389 U/L	<140 U/L
hs-troponin	1577 ng/L − >1619 ng/L − >1538 ng/L	<11 ng/L
NT-pro BNP	2427 pg/mL	<124 pg/mL
CK	29,200 U/L	<355 U/L
CK-MB	525 ng/ml	<6.7 ng/mL
Serum aldolase	130 IU/L	<7.6 IU/L
ESR	85	<30
HMGCR-Ab	59	<20
ANA	Negative	Negative
ASMA	Negative	Negative
Anti-Jo1 antibodies	Negative	Negative

AST: aspartate aminotransferase; ALT: alanine transaminase; ALP: alkaline phosphatase; NT-pro BNP: N-terminal probrain natriuretic peptide; CK: total creatine kinase; CK-MB: creatine kinase-myocardial band; ESR: erythrocyte sedimentation rate; HMGCR-Ab: *β*-hydroxy *β*-methylglutaryl-CoA reductase antibody; ANA: antinuclear antibodies; ASMA: antismooth muscle antibodies.
